# Calcium Binding Mechanism of Soybean Peptide with Histidine Alteration by Molecular Docking Analysis and Spectroscopic Methods

**DOI:** 10.3390/foods11203290

**Published:** 2022-10-20

**Authors:** Jing Gan, Xiao Kong, Ziqun Xiao, Yuhang Chen, Mengdi Du, Yan Wang, Zhenhua Wang, Yongqiang Cheng, Bo Xu

**Affiliations:** 1 Center for Mitochondria and Healthy Aging, College of Life Science, Yantai University, Yantai 264000, China; 2 Beijing Key Laboratory of Functional Food from Plant Resources, College of Food Science and Nutritional Engineering, China Agricultural University, Beijing 100083, China; 3 School of Food Science and Technology, Jiangnan University, Wuxi 214122, China

**Keywords:** histidine, soybean peptide, binding mechanism, molecular docking analyses, spectroscopic methods

## Abstract

Histidine (His) carries a unique heteroaromatic imidazole side chain and plays an irreplaceable role in peptides and proteins. With the current study, we aimed to determine the characteristics and functional activities of the bone density of soy peptide–calcium complexes in which a His residue was replaced by Leu (CBP-H). Soybean peptide (CBP-H) was chemically synthesized, the binding mechanism between CBP-H and calcium ions in combination was determined with bioinformatics and spectroscopy analysis, and the difference between CBP and CBP-H was investigated. Finally, we analyzed the effect of CBP and CBP-H on osteoblasts in vitro. The results showed that CBP-H could bind to calcium ions, and the calcium coordinated with the carboxyl groups of Asp and Glu in the peptide. The nitrogen atoms of the amino group and the oxygen atoms of the carboxyl group in CBP-H significantly contributed to the coordination with Ca^2+^. Furthermore, the binding capacity was 36.48 ± 0.09 mg/g, similar to CBP. However, both CBP and CBP-H could promote osteogenic activity, the activity of CBP-H was 127.147%, lower than CBP (121.777%). While it had the same ability to promote intracellular calcium concentration, CBP-H could upregulate 150.12% calcium ions into the intracellular, and the rate of the rise of CBP was 158.91%, further highlighting the potential of His residues for binding calcium and treating osteoporosis.

## 1. Introduction

Peptides are critical bio-molecules of the human body. They act in living systems, immune regulation, and metabolism. Traditionally, naturally occurring peptides have long been regarded as therapeutic agents and used as a source of new drug development for a long time. For example, casein phosphor-peptides (CPPs), duck egg peptide (VSEE), lactoferrin-derived peptide (PKSETKNLL), soy peptide (DEDEQIPSLPPR), and collagen-binding motif peptide (HHGDQGAPGAVGPAGPRGPAGPSGPAGKDGR; Gly-Ala-Asn-Gly-Asp-Arg-Gly-Glu-Ala-Gly-Pro-Ala-Gly-Pro-Ala-Gly-Pro-Ala-Gly-Pro-Arg) have been proven to enter cells to promote bone proliferation and differentiation [1,2]. However, recently, peptides have served as calcium carriers, which can be directly embedded into the cell membrane to transport calcium ions to the cytosol, promoting calcium bio-availability at a fast transportation rate with low energy consumption, thus resulting in a high absorption. For instance, calcium-binding peptides from sunflower seeds, peanuts, tilapia bone collagen hydrolysate, casein, and Alaska pollock skin have been reported to enhance calcium transportation in Caco-2 cells [3,4,5,6,7,8]. As reported previously, the 20 natural amino acids are the building blocks of peptide structures. Each has its unique structural characters and physicochemical properties and plays an irreplaceable role in biochemistry. The conformation of the polypeptide is the result of the interaction of amino acids. Liu found that the more hydrophobic amino acids in the peptide, the higher the binding capacity with calcium [9]. Meanwhile, the acidic amino acids in the hydrolyzed soybean peptides play a vital role in the absorption rate of calcium [10]. Compared with the high calcium binding capacity produced by the aggregation of long-chain peptides, the short peptide chains are more easily absorbed due to minor steric hindrance.

Among the 20 natural amino acids, histidine (His) plays an important role in the peptide bioactivities [11]. The multiple functions of histidine in molecular interactions are triggered by distinctive molecular structures. The side chain imidazole of histidine is an aromatic motif, which can bind with metal cations such as Zn^2+^ and Ca^2+^ and other amino acids through cation–π interactions, π–π stacking interaction, hydrogen–π interaction, and coordinate bond interaction [12]. The nitrogen atom on the imidazole ring has a strong metal ion affinity compared to the Π-face, which is favorable for binding metal ions in the plane [13]. The binding affinity of histidine to Zn^2+^ and Ca^2+^ was greatly higher than Na^+^ and K^+^, and could also bind to host proteins by coordinate bonds [14]. In addition, the imidazole group of histidine (His) can combine with the carboxyl group in glutamic (Glu) and aspartic (Asp), affecting the structural characteristics of peptides [15]. In comparison, the carboxyl oxygen and amino nitrogen atoms of Glu and Asp are often regarded as the calcium-binding sites in polypeptides. Additionally, the histidine in the molecular showed a positive charge (pK = 6.5), which could reduce the electrostatic repulsion in solution. Thus, targeting His is theoretically an attractive strategy for peptide modification. For example, Huang et al. isolated and purified a high calcium affinity peptide Thr-Cys-His from shrimp [16]. Recently, we purified a novel soy peptide CBP (DEDEQIPSHPPR) with a high calcium affinity and osteogenesis activity, which has one His residue [17]. However, the influence of histidine on the interaction between peptides and calcium is still unclear.

To further investigate the binding mechanism of peptide and calcium, His was replaced with Leu in CBP (CBP-H) (DEDEQIPSLPPR), UV spectroscopy, Fourier infrared spectroscopy (FTIR), X-ray diffraction spectrum (X-ray), isothermal titration calorimetry (ITC), and molecular docking were used to analyze the interaction between CBP-H and calcium. Thermogravimetric analysis-differential scanning calorimetry (TG-DSC) and Caco-2 cells were used to analyze the stability of CBP-H–Ca. Furthermore, the effect of CBP-H on the cell viability of MC3T3-E1 cells was detected. Finally, the effect of CBP-H on bone mass in vivo by the zebrafish osteoporosis model was detected in this study.

## 2. Materials and Methods

### 2.1. Synthesis of Soy Peptides (CBP-H)

Polypeptide CBP (DEDEQIPSHPPR), in which a His residue is replaced by Leu (CBP-H), was synthesized by Nanjing Peptide Industry Biotechnology (Nanjing, China). High-performance liquid chromatography (HPLC) analysis was carried out to determine the purity (98%) of CBP-H, and ESI mass spectrometry was used to confirm the peptide structure. An Agilent MALDI-TOF-MS/MS spectrometer was used to identify the eluted fractions individually (4800, SCIE). An Agilent 1100 HPLC system, electrospray ionization (ESI) interface, and an Agilent 1100 HPLC system were installed, together with the spectrometer. An automatic sampler was used to inject the sample into a C18 trap column (C18, 3 μm, 0.1020 mm), followed by injection into a C18 column (C18, 1.9 μm, 0.15120 mm). Elution was conducted with the aid of solution A (0.1% formic acid in H_2_O) and solution B (0.1% formic acid in acetonitrile) using a gradient of 5–95% B. The flow rate was set at 600 mL/min for 78 min. The peptide sequences were detected using a Q-Exactive mass spectrometer after the separation.

### 2.2. Molecular Docking

The Desmond program was used to perform a molecular docking simulation of the DEDEQIPSHPPR interaction with calcium, combined during molecular docking [18]. The peptide was set up using Maestro 11.8 in the Schrodinger Suite 2018-4, protonated using Epik, and the energy minimization was set at pH 7.0. The solvent system was then developed using the SPC model, in which a minimum distance of 10 Å × 10 Å × 10 Å was placed around the peptide in each direction, and 0.75 mol/L Ca^2+^ was added. Meanwhile, chloride ion counterions were injected into the system to equalize the charge before the energy of the hybrid system was minimized based on the OPLS_3E force field. The simulation process can be divided into six steps: Step 1, Brownian dynamics NVT, T = 10 K, small time steps, and restraints on heavy solute atoms, 100 ps; Step 2: NVT, T = 10 K, small time steps, control of heavy solute atoms, 12 ps; Step 3: NPT, T = 10 K, and restraints on heavy solute atoms, 12 ps; Step 4: NPT, with restraints on solute heavy particles, 12 ps; Step 5: NPT, no restraints, 24 ps; and Step 6: NPT, no restraints, 100 ns. Subsequently, the visual system was visually detected and analyzed

### 2.3. Analysis of the Calcium-Chelating Activity

#### 2.3.1. Preparation of the CBP-H–Ca Complexes and Determination of Calcium Binding

##### Capacity

Peptide–calcium chelate (CBP-H–Ca) was performed according to the method of Cui with slight modification [3]. First, the CBP-H was dissolved in 10 mL of distilled water to a concentration of 10 mg/mL. After that, CaCl_2_ was added to the peptide with a molar ratio of 2:1, and the pH was adjusted to 8.0 with 1 M NaOH. The reaction mixture was mixed in a shaking water bath at 140 rpm and 37 °C for 20 min, and then 90 mL of absolute ethanol was added to the reaction mixture. Finally, the reaction mixture was centrifuged for 10 min at 10,000 g. The precipitate was collected, freeze-dried, and labeled as CBP-H–calcium complexes.

The content of Ca in the CBP-H–calcium complexes was determined by inductively coupled-atomic emission spectrometry (ICP-AES). The detection conditions were as follows: generator power: 1.4 KW, cooling gas flow: 12 L/min, auxiliary gas flow: 0.8 L/min, carrying gas flow: 0.8 L/min.

#### 2.3.2. Isothermal Titration Calorimetry (ITC)

Isothermal titration calorimetry measured the thermodynamic parameters of CBP-H binding with calcium. Both the CBP-H and the CBP-H–Ca were dissolved in Tris-HCl buffer solution to a concentration of 2.5 mM. The sample was degassed, followed by filtration through a 0.22 µm filter. A total of 2.5 mM of CBP-H was loaded into the ITC cell, and the CaCl_2_ solution (50 mM) was put in the ITC syringe. During the measurements, 20 drops of CaCl_2_ solution were injected into the sample cell. The titration parameter was configured to inject CaCl_2_ every 5 min with volumes of 2.5 µL. Nano Analyze software (TA Instrument-Waters LLC, New Castle, DE, USA) was applied to evaluate the raw data based on data fitting [19].

#### 2.3.3. Ultraviolet Spectroscopy

The ultraviolet spectra of the CBP-H and CBP-H–Ca complexes were monitored over the wavelength range from 190 nm to 400 nm with an ultraviolet spectrophotometer (UV-1200, Xiaofen Instrument Co. Ltd., Guangzhou, China). For the determinations, 0.02 mg/mL of peptide was dissolved in MOPS solution (pH = 8.0). After that, 0.2 mM, 0.4 mM, 0.6 mM, and 0.8 mM of CaCl_2_, which also dissolved in MOPS solution, was continuously shaken at 50 °C for 1 h to obtain a peptide–calcium chelate and the UV spectra were recorded.

#### 2.3.4. Fourier Transform Infrared Spectroscopy

The FTIR spectra were recorded using an infrared spectrophotometer (IS50, Thermo Nicolet Co., Waltham, MA, USA) in the range of 4000–400 cm^−1^ at a resolution of 4 cm^−1^, over 32 consecutive scans. In advance of analysis, 3 mg of lyophilized powdered samples and 200 mg of dry KBr were adequately mixed and tableted at 30 MPa [20].

#### 2.3.5. X-Ray Diffractograms

X-ray diffraction (XRD) analysis of lyophilized samples was carried out using a diffractometer (Bruker D8 Advance, Bruker AXS GmbH, Karlsruhe, Germany) equipped with CuKα radiation. The date of diffractograms was recorded for the angle (2θ) ranging from 5 to 85° at a scanning rate of 4°/min, and operating at 40 kV and 40 mA.

### 2.4. Detection of Stability of CBP-H–Ca Complexes

#### 2.4.1. Simulated Gastrointestinal Digestion

The simulation of gastrointestinal digestion was carried out following the method described before with some modifications [21]. The gastric set was reproduced by the suspension of porcine pepsin (40 mg) into 1 mL 0.1N HCl. The intestinal stage was stimulated by adding bile salts (40 mg) and trypsin (10 mg) into 5 mL of 0.1 mol/L NaHCO_3_. Gastric digestion was performed by incubating the CBP–calcium complex or CaCl_2_ (prepared with equal Ca^2+^ concentration, pH = 2) at a pepsin/substrate ratio of 1:100 (*w*/*w*) in a water bath shaker (37 °C, 90 min). During the gastric step, the pepsin at 1:25 (*w*/*w*) of substrates was added, following by adjusting the pH to 7.5. After that, samples were removed after deactivating trypsin by thermal denaturation for 5 min. Then, the obtained samples were lyophilized and stored at −20 °C for the sake of subsequent studies.

#### 2.4.2. Thermogravimetric Analysis -Differential Scanning Calorimetry (TG-DSC)

The thermal decomposition of the peptide–calcium chelate was characterized by differential scanning calorimetry (DSC) and thermogravimetric analysis (TGA) (STA449C, NETZSCH, Germany). The lyophilized samples of about 5 mg were weighed and heated from 40 °C to 600 °C, the programmed heating rate was 20 °C/min, and nitrogen air at 30 mL/min.

#### 2.4.3. Cell Culture and Establishment of Caco-2 Cell In Vitro Absorption Model

The human intestinal Caco-2 cell line has been extensively used as an intestinal barrier model. These cells were cultured in high-glucose Dulbecco’s Eagle’s medium (DMEM), which contained 10% fetal bovine serum (FBS), 100 μg mL^−1^ streptomycin, and 100 U mL^−1^ penicillin, and were incubated at 37 °C in a humidified 5% CO_2_ incubator. Transepithelial transport through the Caco-2 single cell layer was performed in accordance with Wang et al.’s method [22]. The Hank’s balanced salt solution (HBSS) without magnesium and calcium was added to rinse the single layer of Caco-2 cells at least twice, followed by preincubation for 30 min at 37 °C using 1.5 mL of HBSS on the basolateral side and 0.5 mL on the apical side. The absorption assays began by using 0.5 mL of the divided sample fractions at a concentration of 0.1 mg/mL and replenishing the HBSS on the apical side. Subsequently, the cells were incubated at 37 °C for 2 h (Table S1). Meanwhile, the samples of the AP and BL sides were collected, and LC-MS/MS was used to detect the peptide composition, respectively. The LC-MS/MS conditions were similar to those presented in Section 2.1.

### 2.5. Proliferation Assay of MC3T3-E1 Cell

The influence of the CBP-H on MC3T3-E1 cell osteoblast proliferation was quantified by using the MTT assay, as previously described by Zhe et al. [23]. Briefly, the MC3T3-E1 cells were seeded into a 96-well culture plate at a density of 2 × 10^3^ cells/well, and incubated at 37 °C (in 5% CO_2_ and 90% humidity) for 24 h. Then, the media were replaced by CBP at concentrations of 0, 0.7, 7, and 70 μM. After a 72 h incubation period, the cells were treated with 10 μL MTT solution (5 mg/mL in PBS) and incubated at 37 °C for 3 h. After that, the medium was removed and 150 μL DMSO was added to each well followed by shaking the 96-well plates for 10 min. Absorption values of each well were measured by a microplate reader (Molecular Devices, San Jose, CA, USA) at 570 nm.

### 2.6. Intracellular Ca^2+^ Concentration ([Ca^2+^]_i_) Measurement

The influence of the CBP-H on intracellular Ca^2+^ concentration was detected by a flow cytometer, according to Zeng et al. s’ method with slight modifications [24]. Almost 1 × 10^5^ per well was cultured in a 6-well plate. After being treated with CBP-H for 24 h, cells were collected, and 2.5 μ mol/L FLou-3/AM was added and incubated for 1 h. The average fluorescence intensity of cells in each group was detected at a 488 nm excitation wavelength and a 530 nm emission wavelength.

### 2.7. Statistical Analysis

All diagrams were drawn using Origin 8.6 software (Origin Lab, Northampton, MA, USA) and Primase 9.0 software (GraphPad Software, San Diego, CA, USA). All indicators were determined at least in triplicate, and the data are presented as the mean value ± SE. The significant differences were analyzed and evaluated using Duncan in SPSS 19.0 software (*p* < 0.05) (SPSS Institute, Chicago, IL, USA).

## 3. Results

### 3.1. Bioinformatics Analysis of the Interaction between CBP-H and Calcium Ion

#### 3.1.1. Stability of the Dynamic Trajectory from RMSD Analysis

**Figure 1:**
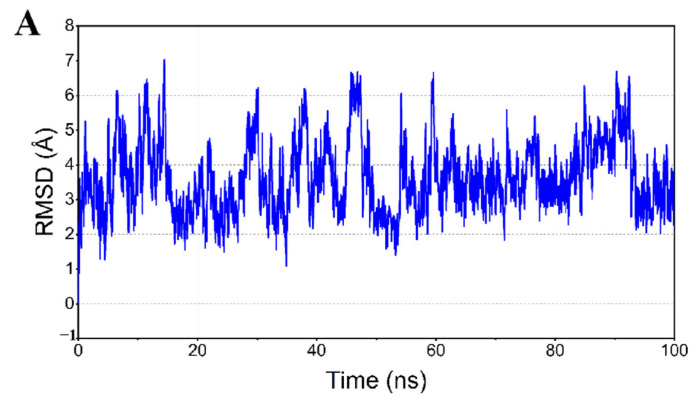

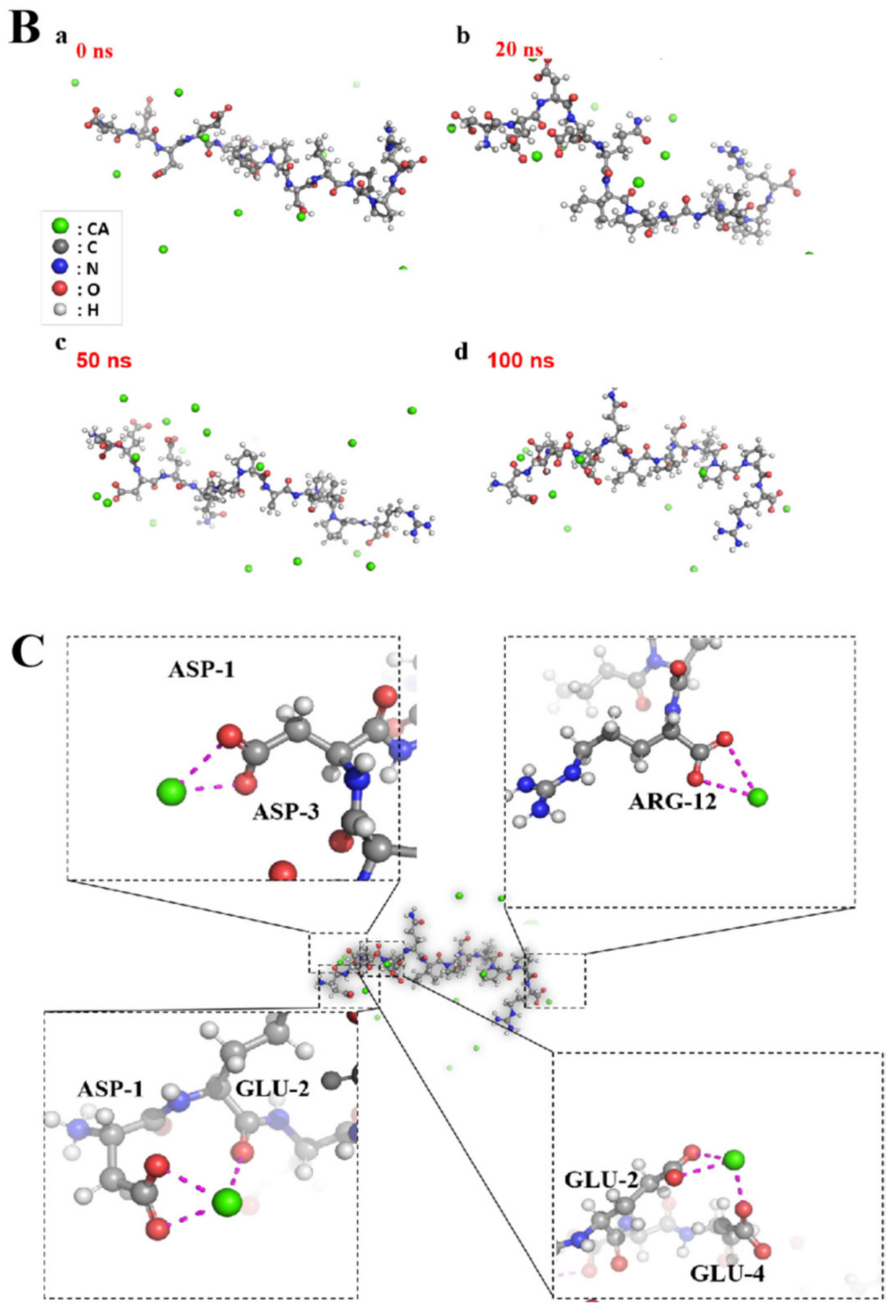
(**A**) RMSD stability of plots of the MD simulations. (**B**) Molecular docking and the structure of DEDEQIPSHPPR-calcium complex at different times (0 ns, 2 ns, 50 ns and 100 ns) of conformations. (**C**) Snapshot of molecular interaction capture at the last frames from the 100 ns MD simulations for the DEDEQIPSHPPR-calcium complex.

The performing molecular dynamics simulations were used to analyze the potential relationship between peptide CBP-H and calcium. In this study, during the simulation process, the total atomic variations of the conformations were used to determine the relative stability of the CBP-H–calcium complex. As shown in Figure 1A, the CBP-H–calcium complex underwent a wide range of 2 A and 6 A fluctuations. However, it was more stable than in a non-Ca^2+^ environment. This indicates that calcium binding is helpful for peptide stability.

#### 3.1.2. Intermolecular Interaction Analysis from MD Simulation

Different conformations (0, 2, 50, and 100 ns) were selected to analyze the interaction between CBP-H and calcium. As shown in Figure 1B, the calcium ions did not interact with CBP-H when the energy of the system was minimized at 0 ns. However, an increasing number of calcium ions bound to peptides over time; for example, three calcium ions can bind to the peptides at 20 ns, and four calcium ions can bind to the peptides at 50 ns, until the binding amount reaches a maximum of five calcium ions at 100 nm. This reveals that the Ca^2+^ ions can completely interact with the peptides and that the saturation number is five.

Intermolecular interaction analysis of the CBP-H–calcium complex during the last frames from the 100 ns MD simulation was performed to compare and clarify the interaction modes and binding type between the CBP-H and calcium. As shown in Figure 1C, the carboxyl group of Glu-2 and Glu-4 in the peptide can bind to two calcium ions via a salt bridge because Glu has a long side chain that can overcome steric hindrance. However, the carboxyl group of Asp-1, Asp-3, and the carboxyl groups of the terminal Arg-12 in the peptide can bind to two calcium ions via charge solvation. Asp-1 and Asp-3 interact with two Ca^2+^ ions through a combination: One Ca^2+^ ion is sandwiched between two acidic amino acids, and the other calcium ion interacts with Asp-1. As a result, Asp-1 might bind to calcium ions in either a bidentate or unidentate mode, under different circumstances. This binding phenomenon stabilizes the conformation of the N-terminus and C-terminus of the peptide.

### 3.2. Analysis of the Calcium-Binding Capacity of the CBP-H by ICP-AES

To clarify the calcium-binding capacity of CBP-H, Nanjing Peptide Biotechnology Corporation. Ltd. (Nanjing, China) synthesized the peptide CBP-H using a solid-phase procedure. High performance liquid chromatography (HPLC) analysis was performed to determine the purity (98%) of CBP-H (Figure S1A), and ESI mass spectrometry was used to confirm the peptide structure. The results showed that the molecular weight of CBP-H was 1395.47 (Figure S1B). In addition, the CBP-H–Ca complex was prepared, followed by ICP-AES to detect the calcium-binding capacity. The results showed that the calcium-binding capacity of the CBP-H was 36.48 ± 0.09 mg/g, which was higher than that of tilapia bone collagen GPAGPHGPVG [25] (18.80 ± 0.49 mg/g), and there was no significant difference with CBP (36.64 ± 0.04 mg/g).

### 3.3. Determination of the Binding Stoichiometry and Binding Constant

**Figure 2:**
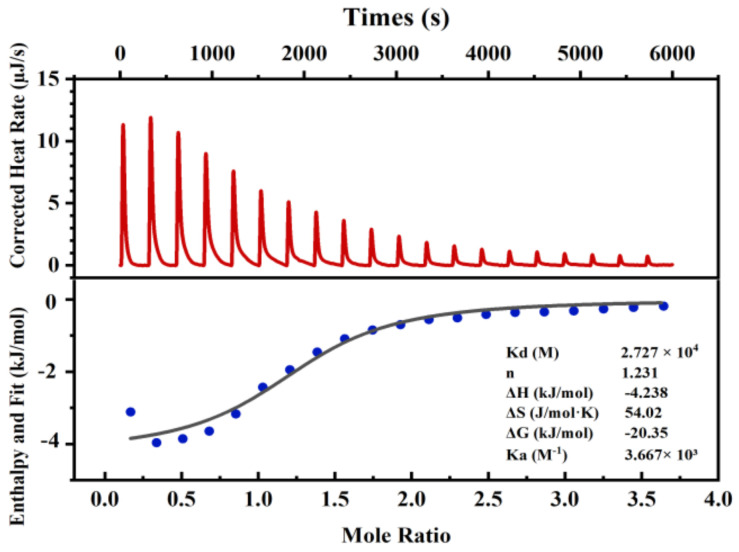
ITC analyses of the CBP-H peptide reacting with calcium ions. The upper panel exhibited a representative calorimetric titration curve. CaCl_2_ (50 mM) was titrated into 2.5 mM of the peptide solution at 25 °C. The lower panel shows the integrated areas corresponding to each titration, plotted as a function of the Ca^2+^/peptide molar ratio. The solid line represents the best curve fit obtained by using an independent binding site model.

In this research, ITC was used to explore the binding amount of calcium to CBP-H. Figure 2 shows a representative calorimetric titration of a CBP-H peptide solution using calcium chloride (CaCl_2_). First, the reference baseline was determined by titrating Tris-HCl buffer (pH = 8.0) with CaCl_2_, followed by 2.5 mM CBP-H solution, and 50 mM CaCl_2_ solution was added to prevent the reaction between the peptides and calcium ions from being incomplete. As shown in Figure 2, this titration resulted in negative ΔH and ΔG values (−4.238 and −20.35 kJ/mol, respectively), demonstrating that the binding reaction was an exothermic, spontaneous process [26]. At the same time, the negative ΔH value also indicated that the heat produced during the coordination bond formation was more significant than the heat absorbed by dehydration during the calcium and peptide binding reactions, accompanied by a positive entropy change (ΔS = 54.02 J/mol·K), which demonstrated that the establishment of the coordination bond caused a more disordered structure [27]. The reason might be related to the peptide side-chain conformational changes or the solvation effect [25]. In addition, considering the entropy change (ΔS >0) and enthalpy change (ΔH <0), the binding mode between CBP-H and calcium can be inferred as ionic interaction forces [28]. Moreover, the reaction stoichiometry (n) and binding constant were 1.23 ± 0.05 and (3.667 ± 0.28) × 10^3^ M^−1^, respectively. This result demonstrates that CBP-H possesses at least one functional calcium-binding location. However, the CBP-H sequence had fewer calcium-binding sites than the CBP, in which the reaction stoichiometry (n) was 1.42 ± 0.04 and (5.23 ± 0.32) ×10^3^ M^−1^, as reported in our previous study.

### 3.4. Functional Groups Responsible for Calcium Binding

**Figure 3:**
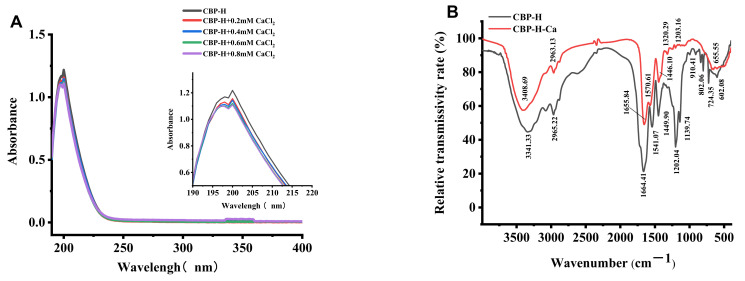
(**A**) UV spectra of CBP-H and CBP-H–Ca with wavelengths in the field of 190–400 nm. (**B**) FTIR spectra of CBP-H and CBP-H–Ca with wavelengths in the range of 190–400 nm.

The UV and FTIR spectra revealed the binding site of the CBP-H–Ca complex. CBP-H showed a strong absorption peak at 200 nm. With an increasing concentration of calcium ions, the absorption intensity decreased from 1.218 to 1.109, with a slight red shift of the maximum absorption wavelength, indicating that a new substance was generated (Figure 3). When the calcium ions bind to the CBP-H, the primary chromophores (–CO, –COOH) and the auxiliary chromophores (–OH, –NH_2_) create polarization change.

To further evaluate the development of the CBP-H–calcium chelate, the FTIR spectra of the CBP-H and the CBP-H–Ca are illustrated in Figure 3, where a remarkable change was found in the CBP-H–Ca chelate after binding with calcium ions. The absorption peak shifted from 3341.33 cm^−1^ to 3408.69 cm^−1^, which may be caused by the interaction of –NH_2_ with Ca^2+^, resulting in N–H stretching and the replacement of the hydrogen bonding with N–Ca. Moreover, the 1449.90 cm^−1^ peaks of CBP-H, which resulted from the symmetric stretching mode of C=O, also shifted to a lower frequency (1446.10 cm^−1^) in the CBP-H–Ca FTIR spectrum. Furthermore, in the fingerprint region, characteristic absorption peaks of the –C–O bond were observed at 1139.74 cm^−1^ and produced a blue shift to 1203.16 cm^−1^, respectively, in the CBP-H–Ca spectrum. In summary, the carboxyl group oxygen atoms and amino group nitrogen atoms in CBP-H play a crucial role in the coordination with Ca^2+^ to form the CBP-H–Ca chelate, which is consistent with previous studies [9] and our previous computer simulation predictions.

### 3.5. Analysis of Structural Change of CBP-H–Ca Complex

**Figure 4:**
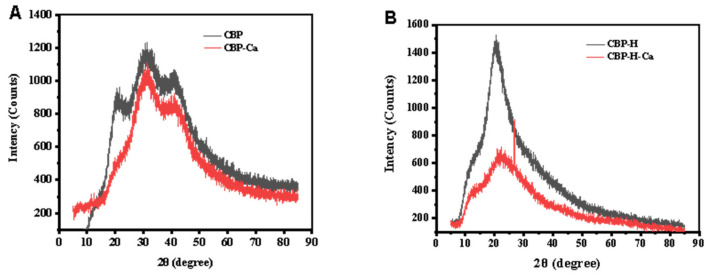
Conformational analysis. (**A**) XRD patterns of CBP and CBP–Ca. (**B**) XRD patterns of CBP-H and CBP–Ca.

The conformational change of CBP-H–Ca is one of the factors affecting the binding strength between calcium and CBP-H. The crystal change was analyzed by XRD. As Figure 4B shows, there were 11 broad and weak dispersion diffraction peaks around 20.5°, 33.9°, 52.5°, 58.6°, and 64.2°, indicating that the CBP is a disordered amorphous structure. With calcium added, the maximum signal peak shifted to near 31.5°, and two faint new diffraction peaks appeared at 12.4° and 47.8°. This phenomenon indicated that a new crystal complex was produced. However, there were only four peaks around 19.7°, 21.4°, 49.4°, and 72.8° for CBP-H, and the derivative peak of CBP-H showed a significant drop after calcium was bound, while a new shapely peak appeared at 26.7°. These results indicate that the mutation of His resulted in a disordered amorphous structure of CBP-H, which was consistent with the previous literature reported by Na Sun [19].

### 3.6. Thermal Stability and Trypsin Digests Stability Analysis of Peptide–Calcium Chelate In Vivo

**Figure 5:**
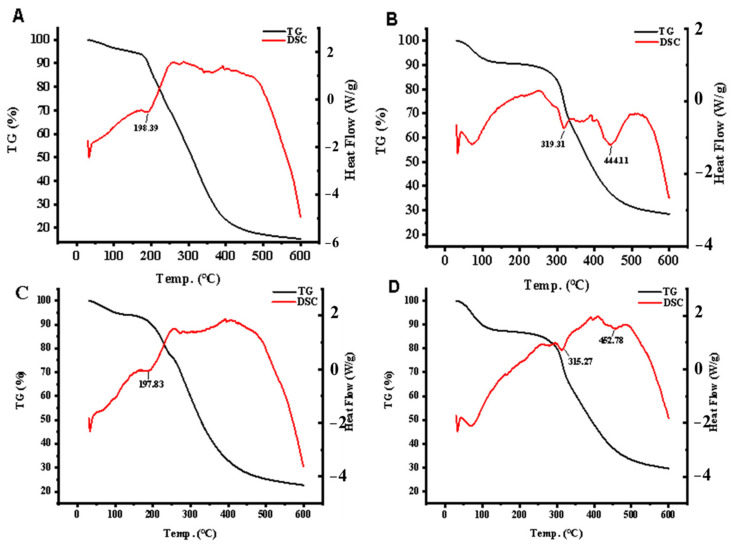
The TG-DSC curves for the peptide and peptide–Ca complexes. (**A**: CBP-H, **B**: CBP-H–Ca, **C**: CBP, **D**: CBP–Ca).

The change in peptide structure will affect its thermal stability and digestibility. Generally, TG-DSC is used to analyze the thermal stability of different components. As shown in Figure 5, there was an obvious thermal stability contrast between the peptide (CBP, CBP-H) and peptide–calcium complexes (CBP–Ca, CBP-H–Ca). Both peptide and peptide–calcium complexes went through three stages: slight degradation stages, major degradation stages, and weak degradation stages. In the slight degradation stages, almost 10% of the peptide amount degraded at 200 °C, which was mainly caused by the adsorption of water on the samples. In the major degradation stage, the majority of proteins was lost. In the weak degradation, only a little amount of protein was degraded until the end of the experiment. In this study, the mass of CBP and CBP–Ca was lost by 72.32% and 59.67%, respectively. Moreover, the endothermic peak of CBP observed at 197.83 °C shifted to 315.27 °C and 452.78 °C under the presence of calcium, which might be caused by destroying C–N bonds during the heating process in different positions. Compared with CBP, CBP–Ca requires a higher fracture temperature. Thus, the stability of CBP–Ca was higher than CBP. In addition, the weight loss of CBP-H and CBP-H–Ca was 80.99% and 64.51%, and the endothermic peak of CBP-H shifted from 198.39 °C to 319.31 °C and 444.11 °C. In summary, the structure of the peptide–calcium complex is more stable than that of the peptide, and the His residues reduce the binding stability with calcium.

To investigate the stability of CBP-H and clarify whether the peptide–calcium complex was completely decomposed during simulated gastrointestinal digestion, LC-MS/MS was used to analyze the CBP, CBP-H, CBP–calcium chelate, CBP-H–calcium chelate, and its simulated trypsin digests after Caco-2 absorption. Table S2 presents the stability analysis of the CBP–calcium chelate, CBP-H–calcium chelate, and its simulated gastrointestinal digests. The complete sequence of the CBP and CBP-H was observed after gastrointestinal digestion, suggesting that these peptides are partially stable. The new sequences DEDQIPSHPPR and EDQIPSLPPR were identified in the digestion product. The *m*/*z* values of the digests was decreased from 710.8283 to 645.2048, and 710.8012 to 581.2893, individually.

### 3.7. CBP-H Stimulated Cell Proliferation in MC3T3-E1 Cells

**Figure 6:**
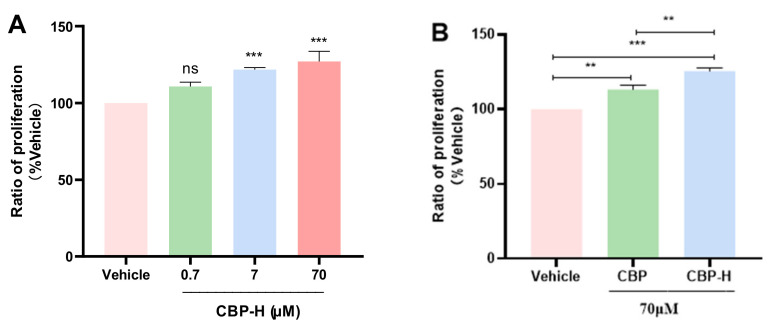
Effects of CBP- H and CBP on cell proliferation. (**A**) CBP-H at concentrations of 0.7, 7, and 70 μM was tested for inducing cell proliferation at72 h. (**B**) CBP-H and CBP at concentrations of 70 μM were tested at 72 h. MTT assays were performed by measuring absorbance at 570 nm. *n* = 5, data are presented as means ± SEMs and analyzed by one-way ANOVA followed by Tukey’s multiple comparison test. ** *p* < 0.01, and *** *p* < 0.001 vs. vehicle.

Peptides can exert functional activity after entering cells through endocytosis. In the previous experiments, we found that CBP could stimulate osteoblast proliferation. To investigate the effect of CBP-H on osteoporosis, we tested the impact of CBP-H on the MC3T3-E1 cell proliferation. As shown in Figure 6, various concentrations (0.7 μM, 7 μM and 70 μM) of CBP-H were added to the culture media for 72 h. Treatment with both 7 μM and 70 μM CBP-H exhibited stimulatory effects on osteoblastic cell proliferation, which were similar to our previous report about CBP. However, the increment rate after peptide mutation was lower than that of native peptides, indicating the importance of His residues for osteogenic activity.

### 3.8. CBP-H Increase the Contents of Intracellular Calcium Ions

**Figure 7:**
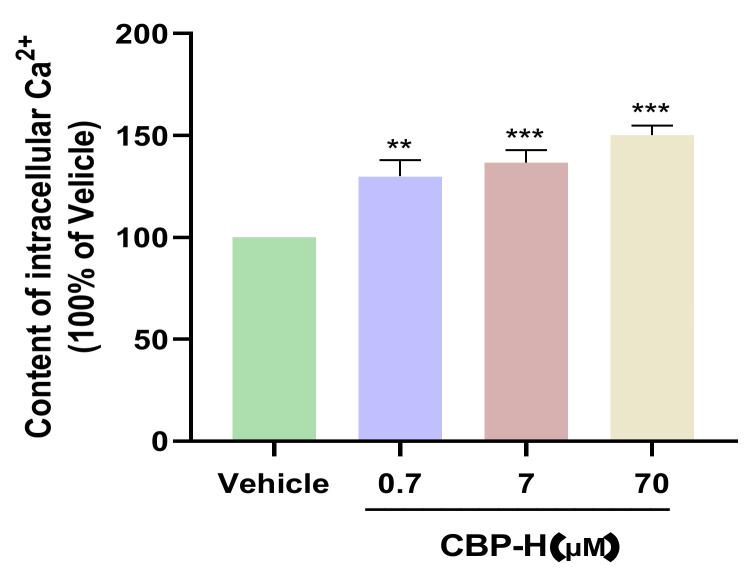
The changes in the intracellular calcium concentration in MC3T3-E1 cells after 0.7, 7, and 70 μM CBP-H treated. ** *p* < 0.01, and *** *p* < 0.001 vs. vehicle.

As the second messenger, calcium plays a crucial role in the process of cell differentiation. In previous studies, it was found that CBP can promote intracellular calcium concentration and thus promote the formation of new bone [17]. To further explore the effect of CBP-H on osteoblasts, flow cytometry analysis was conducted. The results showed that CBP-H could upregulate calcium ions in the cytoplasm, similarly to CBP at 70 μM (158.91%) in our previous research (Figure 7).

## 4. Discussion

The amino acid composition of peptides affects the binding activity with calcium. In our previous study, we purified one soybean peptide CBP (DEDEQIPSHPPR) from soy yogurt that had a high ability to bind calcium and promote osteoblast differentiation. Interestingly, the current published literature has pointed out that histidine residues play a crucial role in peptide function due to its special structure. To assess the roles of His residues in peptides to bind with calcium, we first replaced His by Leu in the peptide CBP, named CBP-H, and investigated the interaction between CBP-H and calcium using molecular docking analysis and spectroscopic methods. Caco-2 cell mode was used to analyze the stability during tryptic digestion and explored the roles of CBP-H in the inhibition of osteoporosis by MC3T3-E1 cells. We found that CBP-H indeed exhibited calcium-chelating capacity and the potential to function as a co-factor in preventing osteoporosis. The nitrogen atoms of the amino group and the oxygen atoms of the carboxyl group in CBP-H significantly contribute to the coordination with Ca^2^. Meanwhile, the Asp and Glu in the carboxyl groups of CBP-H were coordinated with calcium. Additionally, there was no significant difference binding capacity between CBP and CBP-H, but the osteogenic activity of CBP-H was lower than CBP. In summary, His residues can affect the structure of peptides and their functional activity, but play a lesser role than Asp and Glu in the interaction with calcium.

Computer simulation could be utilized to seek active small molecules that fit the binding sites of the receptor [29,30]. In this study, calcium was regarded as a ligand, while the CBP and CBP-H as receptors, respectively. We found that both CBP and CBP-H could bind with calcium ions and had the same binding site, but the binding force of CBP-H (17.624 Kcal/mol) was lower than CBP (23.206 Kcal/mol). It is prominent that CBP presents a globular shape, whereas CBP-H exhibited a linear shape at the C-end, which reduces the binding sites of the peptides with calcium ions (Figure S2A). In addition, Asp and Glu in the peptide play essential roles during the interaction. However, it is unclear whether the replacement of Asp residues with His would affect the binding capacity We replaced the Asp in the CBP and changed CBP (DEDEQIPSHPPR) to DEDHQIPSHPPR and DHDHQIPSHPPR. Meanwhile, the Ser was replaced to His and changed DEDEQIPSHPPR to DEDEQIPHHPPR. The result showed that the -CDOCKER energy of DEDHQIPSHPPR and DHDHQIPSHPPR was lower than CBP and CBP-H, indicating that Asp is more important than His in binding calcium (Figure S1B). The binding force of DEDEQIPHHPPR is higher than CBP-H, but to a lower degree than that for the peptide containing Ser. Next, ICP-AES was used to analyze the calcium ability in CBP and CBP-H. However, there was no significant difference between CBP and CBP-H, where the binding capacity was 36.64 ± 0.04 mg/g and 36.48 ± 0.09 mg/g individually. This may be due to two reasons, on one hand, the nitrogen atom on the imidazole ring in histidine can have an affinity with calcium, which could decrease the binding capacity after His was replaced with Leu. On the other hand, histidine will combine with aspartic acid and glutamic acid to change the spatial structure of peptide, and histidine will present a positive charge in solution, which will decrease the binding capacity by competing for the combination of aspartic acid or glutamic acid with calcium, thus, the binding capacity could increase during His when changed. For the two comprehensive reasons, there was no significant difference. In summary, the content and position of His in peptide will affect the binding mode of peptide with calcium, but the affinity of Asp and Glu with calcium are higher than His.

To verify the reliability of results regarding the binding mode and binding site between CBP-H and calcium, we conducted computer stimulations. Spectroscopic methods were used to analyze the binding mode and binding sites including ITC, UV, FTIR, and XRD. Isothermal titration calorimetry was always used to measure reversible reactions between bio-molecules. This methodology provided the affinity constant, stoichiometry, enthalpy, and entropy of the biomolecular interactions. According to the results of ITC, we found that the CBP-H sequence had numerous calcium-binding sites, but lower than CBP. This is consistent with the computer’s prediction. Moreover, the FTIR showed that the CBP-H can bind calcium by binding carboxy oxygen and amino nitrogen. However, the conformational analysis by XRD revealed that the diffraction peak decreased and there was a sharp diffraction peak after changing the amino acid of His in CBP. It has been reported that the imidazole ring of histidine-containing peptides plays an essential role in metal ion binding [31,32]. The specific calcium-binding peptides from shrimp protein hydrolysates (Thr-Cys-His) exhibited a high calcium binding capacity reaching 2.7 mmol/g, similar to CPP [33], and were confirmed to contain His. The calcium-chelating activity of these peptides could be attributed to their specific groups including the δ-N in the imidazole ring. Meantime, His is not only dependent on the stacking of the cyclic side chain, but the nitrogen atom of the imidazole ring can also act as a hydrogen bond donor and receptor under different conditions with calcium ions [34,35]. In general, peptides with the His residue replaced by other amino acids could bind calcium ions, but the binding capacity was significantly lower than Asp and Glu.

The thermal stability and acid-resisting ability of peptide–calcium complexes affect the bioavailability of calcium. We analyzed the stability of CBP-H using TG-DSC. The results showed that CBP-H had better pH resistance, but lower acid-resisting ability than CBP, implying that His plays a vital role in the conformation of the peptide–calcium complex. To distinguish the stability difference between CBP and CBP-H, the complex and its simulated gastrointestinal digests from Caco-2 cells were evaluated by LC/MS. The initial CBP and CBP-H were also identified in digests, and new other sequences were also identified from the digests. Generally, Leu, as a hydrophobic amino acid, is regarded as the hydrophobic site, but CBP-H was not cut-off at Leu, which is consistent with Cui’s reported data [25]. Although the initial CBP and CBP-H was partially hydrolyzed and freshly generated, it still contained Glu, Arg, and Asp residues and had the capacity to bind to Ca^2+^ ions. However, Glu (E) in the CBP chain was not cut-off. This result is consistent with the results of previous studies, showing that dipeptidyl peptidase IV can create hydrogen bonds with sea cucumber ovum-produced heptapeptides and egg white-simulated digestion products, which can suppress the functioning of dipeptidyl peptidase IV via a charge and π–π interaction mechanism

The functional activity changes of peptides could reflect the effect of His modification on peptides. We analyzed the osteogenic activity of CBP-H and found that CBP-H could promote the proliferation of osteoblasts in a dose-dependent manner. However, the inhibited rate of CBP-H was lower than CBP, which may be due to His being able to produce an affinity with the head of the cell phospholipid molecule, improving the binding with membrane receptors and thus promoting osteogenic activity.

Finally, Ca^2+^, as a messenger in the cells, plays an important role in many physiological processes such as initiating, regulating, and promoting osteoblast proliferation. According to the MTT experiment, we found that both CBP and CBP-H have potential osteogenic activity. To investigate the effect of CBP-H on osteogenic activity, we detected the intracellular calcium concentration. CBP-H can upregulate the intracellular calcium concentration, and the increase ratio was similar to CBP, suggesting that both CBP and CBP-H may promote osteogenic activity by binding with receptor protein in the cell surface and entering the cytoplasm through endocytosis.

## 5. Conclusions

His residence in CBP is critical for binding calcium. The calcium ion can coordinate with the Asp and Glu of carboxyl groups in the peptide. The nitrogen atoms of the amino group and the oxygen atoms of the carboxyl group in CBP-H significantly contribute to the coordination with Ca^2+^. However, further studies are warranted to focus on the mechanisms underlying the anti-osteoporosis effects of amino composition.

## Data Availability

All related data and methods are presented in this paper and the Supplementary Materials. Additional inquiries should be addressed to the corresponding author.

## Supplementary Materials

The following supporting information can be downloaded at: https://www.mdpi.com/article/10.3390/foods11203290/s1, Table S1. The procedures of the absorption washing champers. Table S2. LC-MS/MS-based identification of the simulated gastrointestinal digests of the CBP, CBP-H, CBP–calcium, CBP-H–calcium complex. Table S3. Sequence, localization, and physicochemical properties of CBP-H–calcium complexes. Figure S1. Identifications of CBP-H by HPLC-MS. (A) The MS spectrum of CBP-H. (B) The MS/MS spectrum of CBP-H. Figure S2. Docking calculations for the interaction of soybean peptide (A: CBP-H. B: CBP) with Ca^2+^.

## Abbreviations

CBP	soy peptide
CBP-H	soy peptide with histidine mutation
DEDEQIPSHPPR	Asp-Glu-Asp-Glu-Gln-IIe-Pro-Ser-His-Pro-Pro-Arg
DEDEQIPSLPPR	Asp-Glu-Asp-Glu-Gln-IIe-Pro-Ser-Leu-Pro-Pro-Arg
DEDEQIPHHPPR	Asp-Glu-Asp-Glu-Gln-IIe-Pro-His-His-Pro-Pro-Arg
DHDHQIPSHPPR	Asp-His-Asp- His -Gln-IIe-Pro-His-His-Pro-Pro-Arg
VSEE	Val-Ser-Glu-Glu
PKSETKNLL	Phe-Lys-Ser-Glu-Thr-Lys-Asn-Leu-Leu
DEDEEIPSHPPR	Asp-Glu-Asp-Glu-Gln-IIe-Pro-Ser-His-Pro-Pro-Arg
HHGDQGAPGAVGPAGPRGPAGPSGPAGKDGR	His-His-Gly-Asp-Gln-Gly-Ala-Pro-Gly-Ala-Val-Gly-Pro-Ala-Gly-Pro-Arg-Gly-Pro-Ala-Gly-Pro-Ser-Gly-Pro-Ala-Gly-Lys-Asp-Gly-Arg
GANGDRGEAGPAGPAGPAGPR	Gly-Ala-Asn-Gly-Asp-Arg-Gly-Glu-Ala-Gly-Pro-Ala-Gly-Pro-Ala-Gly-Pro-Ala-Gly-Pro-Arg
