# Supplementary material for: Calcium Binding Mechanism of Soybean Peptide with Histidine Alteration by Molecular Docking Analysis and Spectroscopic Methods
Source: Foods. 2022 Oct 20;11(20):3290. doi: 10.3390/foods11203290 (PMC9601806; doi:10.3390/foods11203290)
Supplement: Supplementary file 1 [file foods-11-03290-s001.zip › foods-1937452-supplementary.pdf]

Supplementary Materials

Table S1. The procedures of absorption washing champers.

Transwell	Procedures		
apical	stop solution 0.5ml/each, three times	removal solution 0.5 ml/each, once	stop solution 0.5ml/each, twice
intermediate	1 N NaOH 0.5 mL/each, twice		
basal	stop solution 1.5mL/ each time, once		

Table S2. LC-MS/MS- based identification of the simulated gastrointestinal digests of the CBP, CBP-H, CBP-calcium, CBP-H-calcium complex.

Sequence	Peptide	Denovo Score	m/z	Conf. (%)
Initial 1	DEDEQIPSHPPR	99	710.8283	99
Initial 2	DEDEQIPSLPPR	99	710.8012	99
Digestion product 1	DEDEQIPSHPPR	99	710.3239	99
Digestion product 2	DEDEQIPSLPPR	99	710.7068	99
Digestion product 3	EDEQIPSHPPR	99	645.8048	99
Digestion product 4	DEQIPSLPPR	99	581.2893	99

Table S3. Sequence, Localization and physicochemical properties of CBP-H-calcium complexes.

AA sequence	-CDOCKER ENERGY	Name	Distance	From	To
DEDEQIPSLPPR	17.624	Ca^{2+}:DEDEQIPSLPPR:O_{56}	2.76934	Ca^{2+}	DEDEQIPSLPPR:O_{56}
		Ca^{2+}:DEDEQIPSLPPR:O_{92}	2.74928	Ca^{2+}	DEDEQIPSLPPR:O_{92}
DEDEQIPSHPPR	23.2059	Ca^{2+}:DEDEQIPSHPPR:O_{56}	2.56309	Ca^{2+}	DEDEQIPSHPPR:O_{56}
		Ca^{2+}:DEDEQIPSHPPR:O_{92}	2.60394	Ca^{2+}	DEDEQIPSHPPR:O_{92}
DEDEQIPHHPPR	21.8751	Ca^{2+}:DEDEQIPHHPPR:O_{56}	2.50702	Ca^{2+}	DEDEQIPHHPPR:O_{56}
		Ca^{2+}:DEDEQIPHHPPR:O_{92}	2.55158	Ca^{2+}	DEDEQIPHHPPR:O_{92}
DEDHQIPSHPPR	12.3839	Ca^{2+}:DEDHQIPHHPPR:O_{70}	2.48884	Ca^{2+}	DEDHQIPHHPPR:O_{70}
DHDHQIPSHPPR	15.8871	Ca^{2+}:DHDHQIPHHPPR:O_{96}	2.57541	Ca^{2+}	DHDHQIPHHPPR:O_{96}
		Ca^{2+}:DHDHQIPHHPPR:O_{121}	2.6225	Ca^{2+}	DHDHQIPHHPPR:O_{121}

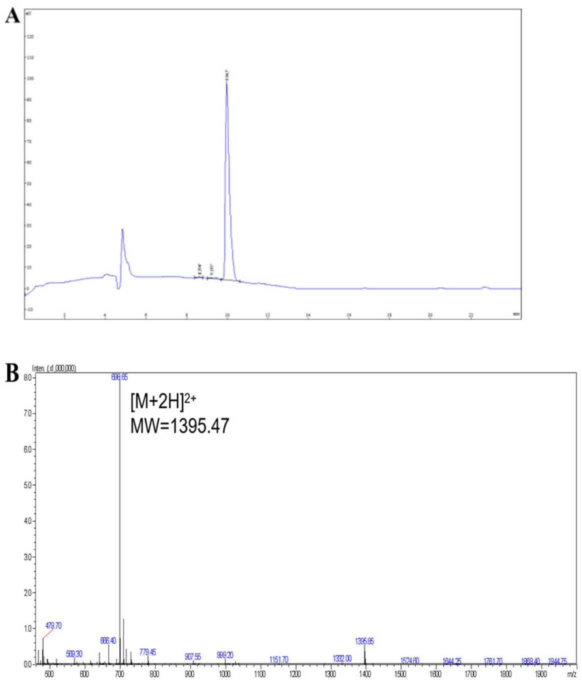

Figure S1. Identifications of CBP-H by HPLC-MS. (**A**): The MS spectrum of CBP-H. (**B**): The MS/MS spectrum of CBP-H.

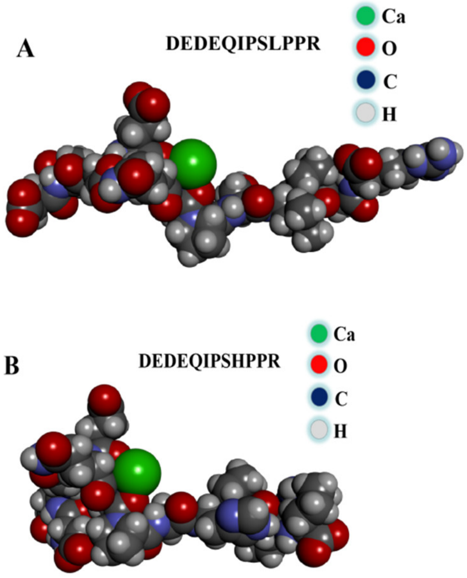

Figure S2. Docking calculations for the interaction of soybean peptide (**A**): CBP-H. (**B**): CBP with Ca^{2+}.
